# Evaluation of the Dermal Toxicity of InZnP Quantum Dots Before and After Accelerated Weathering: Toward a Safer-By-Design Strategy

**DOI:** 10.3389/ftox.2021.636976

**Published:** 2021-03-22

**Authors:** Fanny Dussert, Karl David Wegner, Christine Moriscot, Benoit Gallet, Pierre-Henri Jouneau, Peter Reiss, Marie Carriere

**Affiliations:** ^1^Université Grenoble Alpes, CEA, CNRS, IRIG, SyMMES, CIBEST, Grenoble, France; ^2^Université Grenoble Alpes, CEA, CNRS, IRIG, SyMMES, STEP, Grenoble, France; ^3^Integrated Structural Biology Grenoble (ISBG), UMS 3518, CNRS, CEA, Université Grenoble Alpes, Grenoble, France; ^4^Université Grenoble-Alpes, CNRS, CEA, IBS, Grenoble, France; ^5^Université Grenoble Alpes, CEA, CNRS, IRIG, MEM, LEMMA, Grenoble, France

**Keywords:** quantum dot, toxicity, genotoxicity, safer-by-design, human primary keratinocyte, skin, environmental aging, end of life

## Abstract

Quantum dots (QDs) are colloidal fluorescent semiconductor nanocrystals with exceptional optical properties. Their widespread use, particularly in light-emitting diodes (LEDs), displays, and photovoltaics, is questioning their potential toxicity. The most widely used QDs are CdSe and CdTe QDs, but due to the toxicity of cadmium (Cd), their use in electrical and electronic equipment is now restricted in the European Union through the Restriction of hazardous substances in electrical and electronic equipment (RoHS) directive. This has prompted the development of safer alternatives to Cd-based QDs; among them, InP QDs are the most promising ones. We recently developed RoHS-compliant QDs with an alloyed core composed of InZnP coated with a Zn(Se,S) gradient shell, which was further coated with an additional ZnS shell to protect the QDs from oxidative surface degradation. In this study, the toxicity of single-shelled InZnP/Zn(Se,S) core/gradient shell and of double-shelled InZnP/Zn(Se,S)/ZnS core/shell/shell QDs was evaluated both in their pristine form and after aging in a climatic chamber, mimicking a realistic environmental weathering. We show that both pristine and aged QDs, whatever their composition, accumulate in the cytoplasm of human primary keratinocytes where they form agglomerates at the vicinity of the nucleus. Pristine QDs do not show overt toxicity to cells, while aged QDs show cytotoxicity and genotoxicity and significantly modulate the mRNA expression of proteins involved in zinc homeostasis, cell redox response, and inflammation. While the three aged QDs show similar toxicity, the toxicity of pristine gradient-shell QD is higher than that of pristine double-shell QD, confirming that adding a second shell is a promising safer-by-design strategy. Taken together, these results suggest that end-of-life degradation products from InP-based QDs are detrimental to skin cells in case of accidental exposure and that the mechanisms driving this effect are oxidative stress, inflammation, and disturbance of cell metal homeostasis, particularly Zn homeostasis. Further efforts to promote safer-by-design formulations of QDs, for instance by reducing the In and Zn content and/or implementing a more robust outer shell, are therefore warranted.

## Introduction

Quantum dots (QDs) are colloidal fluorescent semi-conductor nanocrystals that show remarkable optical properties such as tunable band gap, broad absorption and narrow emission spectra, high quantum yield (QY), and resistance to photobleaching (Reiss et al., 2016; Jang et al., 2020). They are currently used in optoelectronic applications, where they are incorporated in displays such as QD-LCD televisions and in light-emitting diodes (LEDs), as well as for biomedical imaging and biosensing (Piccinno et al., 2012; Wegner and Hildebrandt, 2015). Over the past few years, a trend toward the development of cadmium (Cd)-free QDs has emerged due to the toxicity and carcinogenicity of this heavy-metal element (IARC, 1993) that led the European Parliament to limit its use via the Restriction of hazardous substances in electrical and electronic equipment (RoHS) directive (2011/65/EU). Indium phosphide (InP) QDs range among the most promising Cd-free RoHS-compliant QDs and show tunable emission from visible to the near-infrared range (Ung et al., 2010; Reiss et al., 2016). Although InP QDs are considered to be a safer alternative to CdSe QDs (Brunetti et al., 2013), InP as a material is classified as probably carcinogenic to humans (IARC, 2006).

Currently, intense research effort is dedicated to the adoption of a safer-by-design approach when preparing new nanomaterials. This approach aims to limit the exposure to a material and reduce its adverse effects on human health and the environment throughout its whole life cycle, from conception to disposal, while preserving its properties (Bottero et al., 2017; Schwarz-Plaschg et al., 2017). A general safer-by-design strategy for QDs is to prevent ion leaching to the environment by epitaxial growth of another semiconductor layer on the QD core, which has a stronger resistance or at least slows down the chemical and photochemical degradation. QD degradation derives from surface oxidation and is accelerated by exposure to ambient air and UV light, and the passivation of the QD core with a shell is limiting the release of byproducts resulting from degradation (Reiss et al., 2016). Regarding InP QDs, shell materials such ZnSe or ZnS were used and proved to limit the degradation and thereby reduced the QD toxicity (Derfus et al., 2004; Tarantini et al., 2019). To further decrease the exposure, the use of low QD concentrations within a device or biological application demands high-quality photophysical properties such as high photoluminescence quantum yield (PL QY). The incorporation of zinc in alloyed InZnP core QDs has shown to significantly increase the PL QY in comparison to InP core QDs (Li and Reiss, 2008; Ung et al., 2010). Moreover, selecting a surface ligand that enhances the QD stability by tuning its surface charge or mixing QDs with lipids also efficiently reduces their degradation and hence their toxicity (Karabanovas et al., 2008; Chen et al., 2018). InP/ZnS QDs have shown to accumulate in the lymph nodes, liver, and spleen of mice and rats exposed by intravenous injection, intratracheal instillation, or subcutaneously (Yaghini et al., 2016, 2018; Li et al., 2020; Lin et al., 2020). Overall, they show mild toxicity on rodents. InP/ZnS QDs coated with polymers terminated by –OH, –NH_2_, or –COOH modulate hematological parameters related to liver or cardiac function when administered intravenously to mice at high dose (Li et al., 2020). They cause hyperemia in alveolar septa when administered by intratracheal instillation (Lin et al., 2020). Conversely, intravenous or subcutaneous injection of 12.2 or 58 nm PEGylated InP/ZnS QDs does not show any sign of inflammation and toxicity to rats or mice (Lin et al., 2015; Yaghini et al., 2016, 2018). *In vitro*, InZnP/Zn(Se,S) QDs degrade when exposed to UV light in environmental conditions (Tarantini et al., 2019). Their degradation leads to the release of In(III)-phosphate and -carboxylate and increases their cytotoxicity toward human primary keratinocytes concomitantly with inhibition of cell proliferation (Tarantini et al., 2019). Pristine InP/ZnS QDs alter the viability of BGC-823 gastric cells, SHSY-5Y neuroblastoma cells, and HCC-15 and RLE-6TN lung cells via the induction of apoptosis (Liu et al., 2015; Chen et al., 2018). They trigger oxidative stress, endoplasmic reticulum stress, and inflammation in mouse bone marrow-derived macrophages (Chen et al., 2019).

These studies underline the need to design more robust QD core/shell structures, which would further enhance the stability of InP QDs, thereby reducing their toxicity. We recently reported the synthesis of safer-by-design InP QDs consisting of an InP core alloyed with Zn (InZnP) and coated with a Zn(Se,S) gradient shell, which was further capped with a thin or thick ZnS shell (Wegner et al., 2019). These double-shelled QDs show better stability when incorporated into a poly(methyl methacrylate) matrix and exposed to UV light, as well as reduced cytotoxicity toward human primary keratinocytes (Wegner et al., 2019). Despite the reduced toxicity, we wanted to gain a better understanding of the mechanisms leading to the toxicity of these newly developed QDs, both in their pristine state and at their end-of-life, i.e., after accelerated weathering in a climatic chamber. We investigated their impact on cell viability, DNA integrity, oxidative balance of human primary keratinocytes, as well as their inflammatory potential. Since the degradation of these QDs leads to the release of In(III) and Zn(II) ions, we explored the impact of the three types of QDs mentioned above on cellular metal homeostasis, particularly Zn homeostasis.

## Materials and Methods

### Chemicals and Reagents

**Figure 1:**
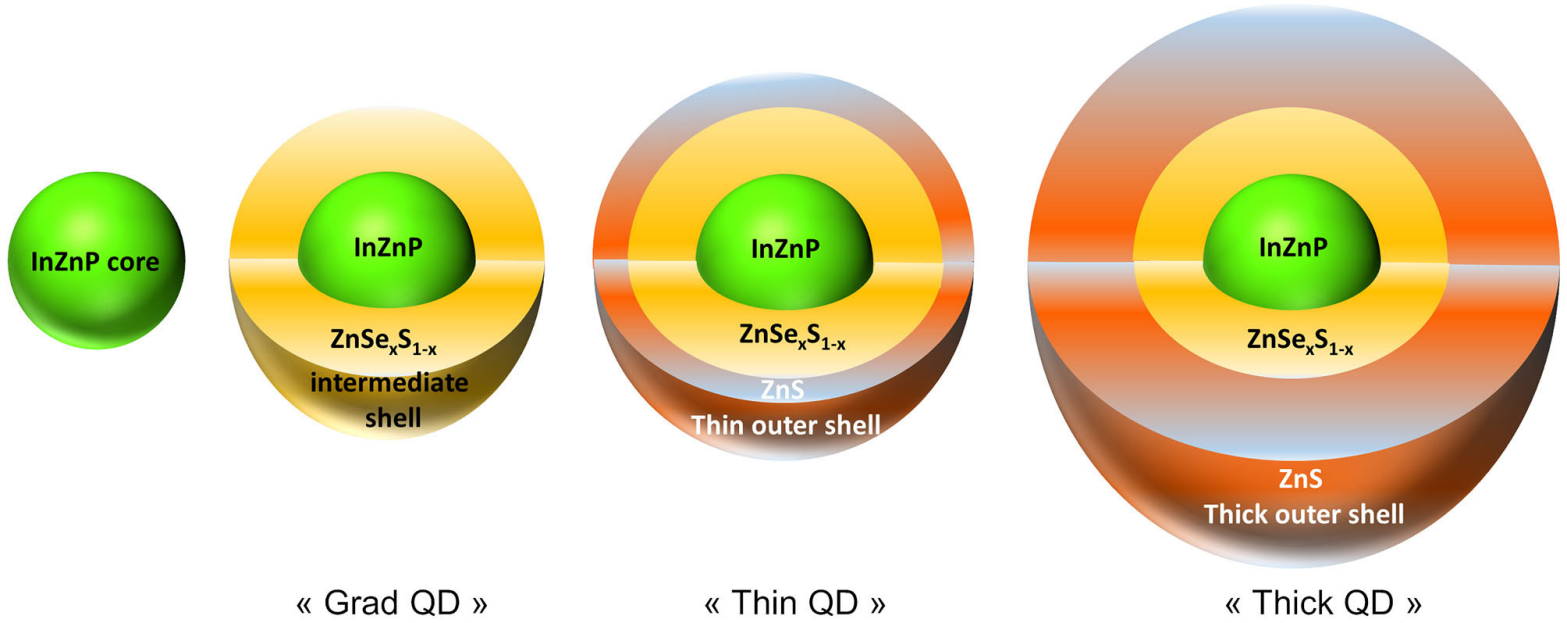
Schematic representation of the quantum dots (QDs) used in the present study. The following QDs were used: (i) QDs composed of an InZnP core coated with a gradient shell of ZnSe_*x*_S_1−*x*_, i.e., Zn(Se,S) (grad QD), (ii) QDs having a double shell where the first shell is a Zn(Se,S) shell and the second shell is a thin outer ZnS shell (thin QD), and (iii) QDs having a double shell where the first shell is composed of Zn(Se,S) and the second shell is a thick ZnS shell (thick QD).

Cell culture medium and supplements were purchased from Thermo Fisher Scientific. All other chemicals and reagents were purchased from Sigma-Aldrich. QDs were synthesized in our laboratories and transferred to phosphate-buffered saline (PBS) by ligand exchange as described previously (Wegner et al., 2019). Three types of QDs were used in this study. The first one was a single-shelled QD, composed of an InZnP core coated with a gradient shell of Zn(Se,S) (referred to as grad QD). Then, two double-shelled QDs were used, in which the gradient shell of Zn(Se,S) is further coated with a thin shell of ZnS (referred to as thin QD) or with a thick shell of ZnS (referred to as thick QD). Figure 1 shows a schematic representation of these QDs. Their photophysical and structural properties are summarized in Supplementary Table 1.

### Accelerated Weathering

During normal use of QD-containing devices, they are exposed to UV light, which degrades the QDs. To simulate such degradation, the InZnP QDs were submitted to an accelerated weathering procedure, in a climatic chamber. QDs were diluted to 1 μM in PBS and 2 ml of these suspensions were placed in quartz cuvettes (10 mm × 10 mm, path length 10 mm) closed by a screw cap with silicone rubber seal. Then, the cuvettes were exposed to full-spectrum sunlight in a Q-SUN Xe-1 Xenon arc test chamber (Q-LAB). The settings of the test chamber were chosen according to the ISO norms 4892-1 (2000) and 4892-2 (2013), which are dedicated to the study of plastic aging in environmental conditions. The rationale for choosing these norms was that these QDs are intended to be incorporated into plastic polymers in optoelectronic applications. The temperature was fixed at 40°C and the irradiance at 1.44 W/m^2^ (measured at 420 nm), without humidity control, as in our previous study (Tarantini et al., 2019).

### Cell Culture and Exposure

Human primary keratinocytes were isolated from skin donors as previously described (Mouret et al., 2006). Skins were obtained from the University hospital of Grenoble from healthy females with their informed consent. Experiments followed guidelines and regulations, in particular the article L1245-2 of the French Public Health Code on the use of surgical wastes for research purposes. Young healthy Caucasian donors were selected for this study (15–30 years old) with phototype I or II according to the Fitzpatrick classification.

After isolation, cells were grown in keratinocyte serum-free medium (KSFM) supplemented with 1.5 ng/ml epidermal growth factor, 25 μg/ml bovine pituitary extract, and 75 μg/ml primocin and maintained at 37°C, 5% CO_2_ in humidified atmosphere. They were used only at the first three passages, and they were passaged at <80% of confluence. They were seeded in clear or black standard 96-well-plates for cytotoxicity assays or reactive oxygen species (ROS) measurements, respectively, at a density of 20,000 cells per well. For 53BP1 immunostaining, they were seeded in black 96-well-plates with clear bottom, at a density of 10,000 cells per well. For comet and quantitative reverse transcription PCR (RT-qPCR) assays, they were seeded at a density of 250,000 cells per well in 12-well-plates. Finally, they were seeded at 120,000 cells per well in four-chamber Labteks for electron microscopy preparations. The day after seeding, cells were exposed to 3.125–100 nM pristine or aged QDs for 24 h or to 25–250 μM of In(II)-acetate or Zn(II)-acetate.

### Transmission Electron Microscopy

After exposure, cells were fixed with 2% paraformaldehyde mixed with 0.2% glutaraldehyde prepared in PHEM buffer (5 mM HEPES, 5 mM PIPES, 10 mM EGTA, 2 mM MgCl_2_, and pH 7), for 30 min at room temperature. The fixative solution was replaced with fresh one, and the samples were incubated for another 30 min at room temperature. Then, cells were post-fixed for 1 h with 1% OsO_4_ and dehydrated in a graded EtOH series (30–100%). Uranyl acetate (0.5%) was added in the first step of dehydration, i.e., in the 30% EtOH solution that was applied for 15 min on the samples. No post-staining was used, in order to avoid the formation of electron-dense precipitates that could make it difficult to identify QDs. Samples were then embedded in EPON resin. Then, 100-nm-thin sections were cut and collected on formvar carbon-coated copper grids, as described previously (Tarantini et al., 2019). Formvar carbon-coated grids were used to avoid any photodegradation of the sample due to the beam irradiation. They were imaged using a Tecnai G2 Spirit BioTwin (FEI) transmission electron microscope (TEM) operating at 120 kV. Images were recorded with an ORIUS SC1000 CCD camera (Gatan). For energy-dispersive X-ray spectroscopy (EDX) analysis, samples were imaged by scanning-transmission electron microscopy (STEM) using a high-angle annular dark field (HAADF) detector, then analyzed on a FEI/Tecnai OSIRIS microscope operating at 200 kV.

### Toxicity Assessment

#### Cell Viability

Cytotoxicity was assessed using the lactate dehydrogenase assay (LDH, Sigma-Aldrich) and WST-1 assay (Roche), which probe cell membrane integrity and cell metabolic activity, respectively. Amino-modified polystyrene nanoparticles (PS-NH_2_) (100 μg/ml) were used as positive control. At the end of the exposure time, for LDH quantification, 50 μl of the exposure medium was transferred to a clean 96-well-plate and mixed with 100 μl of LDH reagent (substrate, cofactor, and dye, vol./vol./vol.). After 30 min of incubation in the dark at room temperature, 10 μl of hydrochloric acid (HCl) 1 N was added into each well to stop the reaction. The absorbance was measured at 490 nm, as well as the background absorbance at 690 nm, using a SpectraMax M2 spectrofluorometer (Molecular Devices). To evaluate the interference of QDs remaining in the exposure medium with this assay (both optical interference and interference with the LDH reaction), LDH reagent was mixed with 100 nM of each QD and this solution was incubated for 30 min at room temperature. Absorbance was measured at 490 and 650 nm with a SpectraMax M2 spectrofluorometer (Molecular Devices). Moreover, the absorbance of QDs at these wavelengths was measured and compared to the LDH test results. These tests did not reveal any interference of QDs (Supplementary Figure 1).

Then, the remaining exposure medium was removed from the wells, and cells were washed with PBS then exposed to 100 μl of WST-1 diluted to the tenth in cell culture medium. After 90 min of incubation at 37°C and 5% CO_2_, absorbance at 450 and 650 nm was measured using a SpectraMax M2 spectrofluorometer (Molecular Devices). To evaluate the interference of QDs with the WST-1 assay, plates were centrifuged and the supernatant of each well was transferred to a clean plate. Again, absorbance was measured at 450 and 650 nm with a SpectraMax M2 spectrofluorometer (Molecular Devices). Values obtained before and after centrifugation did not significantly differ; therefore, we considered that there was no optical interference of the QDs with the WST-1 assay. To evaluate the potential interference of QDs with the chemical reaction leading to the formation of the colored formazan product, WST-1 was mixed with 100 nM of QD. After 90 min of incubation at 37°C, absorbance was measured at 450 and 650 nm with a SpectraMax M2 (Molecular Devices). This test did not reveal any interference of QDs with the WST-1 reaction (Supplementary Figure 1). WST-1 and LDH experiments were repeated three times independently, on keratinocytes from different donors, with *n* = 5 replicates in each independent experiment.

#### Reactive Oxygen Species Intracellular Content

ROS content was quantified by fluorescence measurements using 2′,7′-dichlorodihydrofluorescein diacetate (H2DCFDA) (Thermo Fisher Scientific). Cells were exposed to 25 μM of H2DCFDA diluted in PBS, then incubated for 40 min at 37°C, 5% CO_2_. They were then washed and exposed to QDs. Hydrogen peroxide (H_2_O_2_, 500 μM) was used as positive control. Fluorescence at λexc/λem 480/530 nm (cutoff 515 nm) was monitored for the next 24 h, i.e., 0, 30 min, 1, 2, 4, 6, and 24 h after exposure, using a SpectraMax M2 spectrofluorometer (Molecular Devices). The fluorescence emission of the QDs at this wavelength, when excited at 480 nm, was negligible (not shown); therefore, we concluded that interference of QDs with this assay would be minimal. Each experiment was repeated three times independently on keratinocytes from different donors with *n* = 5 replicates for each of independent experiment. This experiment was also performed on cells exposed to In(III)-acetate or Zn(II)-acetate, but using the dihydrorhodamine 123 dye (DHR123, Thermo Fisher Scientific), which is another probe of ROS intracellular content, with the same experimental procedure.

#### Damage to DNA

Double-strand breaks in DNA were quantified by 53BP1 immunostaining and foci counting, as described previously (Dorier et al., 2019). After seeding, cells were exposed for 24 h to QDs. Etoposide (100 μM) was used as positive control. After exposure, cells were fixed in 4% paraformaldehyde for 30 min at room temperature and then permeabilized for 15 min with 0.2% Triton X-100 prepared in PBS containing 3% bovine serum albumin (PBS-BSA). Non-specific sites were blocked for 15 min in PBS-BSA. Cells were then incubated for 1 h with rabbit polyclonal anti-TP53BP1 antibody (Abnova, reference PAB12506) diluted in PBS-BSA. They were washed three times for 5 min with PBS-BSA and then incubated for 1 h with an anti-rabbit IgG Atto 633 antibody (Sigma-Aldrich, 41176) diluted in PBS-BSA. This secondary antibody was chosen because no QD fluorescence was expected at this wavelength (deep red), avoiding any interference with the automatic counting of 53BP1 foci. Finally, cells were washed three times for 15 min with PBS-BSA to which 0.2% Triton X-100 was added, and their nuclei were stained with 0.3 μg/ml Hoechst 33342 (Sigma-Aldrich) for 20 min at room temperature. The plates were stored at 4°C in PBS containing 50% glycerol until analysis using a CellInsight CX5 High-Content Screening (HCS) Platform (Thermo Fisher Scientific). The number of cell nuclei and the average number of 53BP1 foci per cell nucleus were counted in each well. These experiments were repeated three times independently, on keratinocytes from different donors, with *n* = 5 replicates for each of independent experiment.

In addition to the 53BP1 assay, the presence of DNA strand breaks and Fpg-sensitive sites was evaluated using the alkaline and Fpg-modified comet assays, as described previously (Armand et al., 2016). Cells exposed for 24 h to 100 μM of methyl methanesulfonate (MMS) were used as positive control. Comet assays were performed with two gels per slides, at a density of 12,000 cells per gel. Analysis consisted in recording the percentage tail DNA on 50 cells per gel using the Comet IV software (Perceptive Instruments, Suffolk, UK). Experiments were repeated six times independently, on keratinocytes from three different donors, with two technical replicates per condition in each independent experiment.

#### mRNA Expression

Quantitative reverse transcription PCR (RT-qPCR) was used to quantify mRNA expression level in cells exposed to QDs. Superoxide dismutase 1 (SOD1), SOD2, glutathione peroxidase (GPX1), catalase (CAT), heme oxygenase (HO-1), and glutamate–cysteine ligase (GCLM) were chosen as probes of oxidative stress; metallothionein (MT)1, MT2, MTF1, ZnT1, ZnT7, ZIP1, and HSPA6 as probes of cellular metal and zinc homeostasis; and IL-8, IL-1β, and TNF-α as probes of inflammation. For normalization, CycloA, GADPH, and S18 were used as reference genes. Primer sequences are reported in Supplementary Table 2. RNA was extracted from keratinocytes using GenElute^TM^ mammalian total RNA miniprep kit (Sigma-Aldrich), following the manufacturer's instructions. RNA purity and concentration were determined by absorbance measurement at 260 nm and calculation of abs. 260/280 nm and abs. 260/230 nm ratios, using a NanoDrop^TM^ spectrophotometer. Then, mRNA were retro-transcribed to cDNA using SuperScript III Reverse Transcriptase (Invitrogen), using 0.5 μg of RNA, 100 ng/μl random primers, 10 mM dNTP mix, and 45 U/μL ribonuclease inhibitor. RT-qPCR was performed using a CFX96 Touch System (Bio-Rad) using SYBR Green SuperMix (Bio-Rad). Values were calculated using the Relative Expression Software Tool (REST2009) (Pfaffl, 2001), based on the ΔΔCq method.

### Statistical Analysis

We used RStudio, the interface of R 3.3.2 software, to assess statistical significance, applying non-parametric Kruskal–Wallis test followed by pairwise comparisons using Wilcoxon rank sum test. When multiple comparisons were performed, *P*-values were adjusted using Benjamini and Hochberg correction, and results were considered statistically significant (^*^) when *p* < 0.05.

## Results

Human primary keratinocytes were exposed for 24 h to QDs coated with a gradient shell composed of Zn(Se,S) (grad QD), a double shell with the thin outer ZnS shell (thin QD), or a double shell with the thick outer ZnS shell (thick QD). The structure and composition of these QDs are illustrated in Figure 1. The physico-chemical characteristics of these QDs are reported in Supplementary Table 1. All of them were tested either in their pristine form or after 24 h of accelerated weathering in a climatic chamber.

### QD Accumulation and Intracellular Distribution

**Figure 2:**
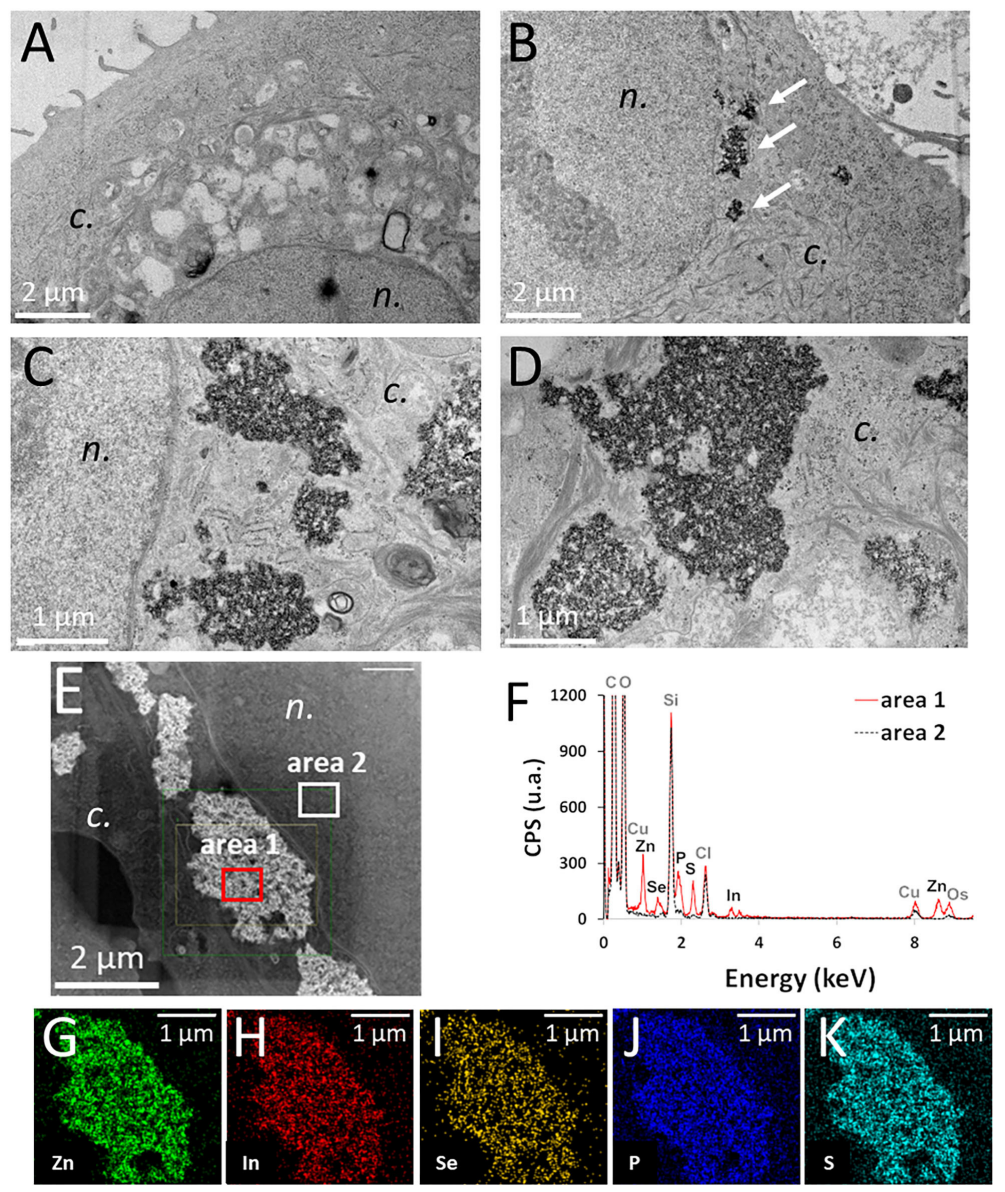
Intracellular distribution of pristine thick shell quantum dots (QDs) in keratinocytes, observed by transmission electron microscope (TEM) and scanning-transmission electron microscope (STEM) imaging, coupled to energy-dispersive X-ray spectroscopy (EDX) analysis. Control cells (unexposed) **(A)**, cells exposed for 24 h to 50 nM of pristine thick shell QD [**(B–D)**, white arrows in panel **(B)**: agglomerates of QDs]. High-angle annular dark-field imaging (HAADF) mapping of a cell exposed to 50 nM of pristine thick-shell QD **(E)** and EDX analysis of the areas 1 (red) and 2 (black dashed) of this image **(F)**, with image representation of the distribution of Zn (**G)**, In **(H)**, Se **(I)**, P **(J)**, and S **(K)**. *n*, nucleus; *c*, cytoplasm.

Figure 2A shows control keratinocytes, not exposed to QDs. In keratinocytes exposed to pristine QDs, electron-dense agglomerates were observed inside the cell cytoplasm, close to the nuclear membrane, both by TEM (Figures 2B–D, arrows in Figure 2B) and by STEM (Figure 2E). Whether these agglomerates were inside cytoplasmic vesicles or not could not be concluded firmly, as no membrane could be distinguished around them, even at high magnification (Figures 2C,D). QD agglomerates were neither located in the endoplasmic reticulum nor in the Golgi, mitochondria, or nucleus. These agglomerates were analyzed by EDX, which proved that they contained In, Zn, Se, S, and P (Figures 2F–K), i.e., that they were agglomerates of QDs. These five elements were colocalized, and no significant ion leaching from the QD material was observed. In keratinocytes exposed to aged QDs, electron-dense precipitates were observed also inside cells (Supplementary Figure 2), although these precipitates were rarely observed. This suggests that QDs degraded and their degradation products coprecipitated, as also observe previously (Tarantini et al., 2019).

### Cytotoxicity of Pristine and Aged QDs

**Figure 3:**
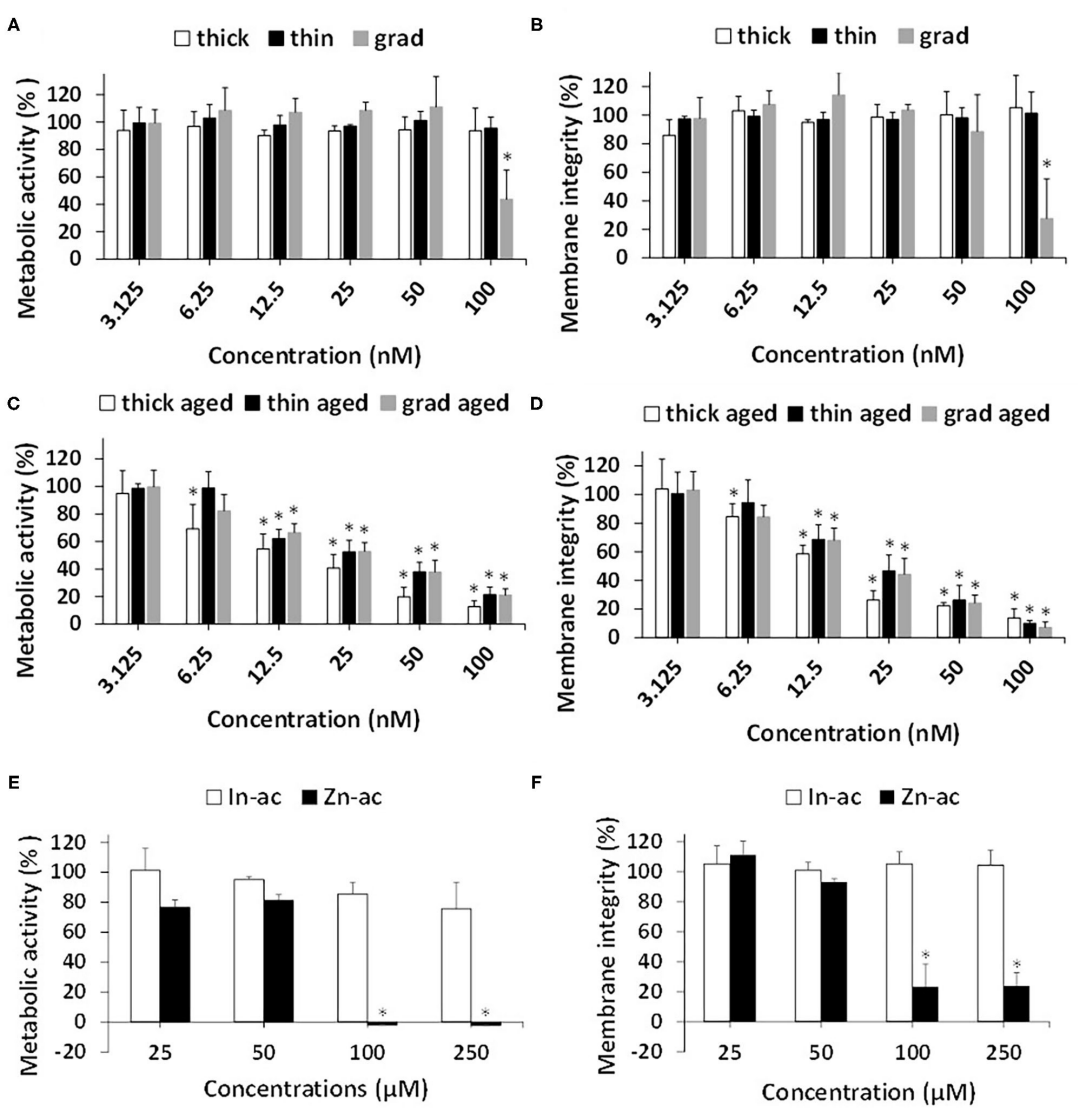
Cytotoxicity of InZnP QDs, In(III)-acetate, and Zn(II)-acetate. Cells were exposed for 24 h to pristine quantum dots (QDs) **(A,B)** or aged QDs **(C,D)** or In(III)-acetate (In-ac) and Zn(II)-acetate (Zn-ac) **(E,F)**. Cytotoxicity was assessed via the WST-1 assay (1) **(C,E)** and lactate dehydrogenase assay **(B,D,F)**. Polystyrene amine (PS-NH_2_) nanoparticles (100 μg/ml) were used as positive control (not shown). Results are expressed as percentage cell viability (metabolic activity or membrane integrity) relative to the control (unexposed cells). Data are mean ± standard deviation of three independent experiments, performed on human primary keratinocytes from three different donors, with five replicates per experiment. Statistical significance: *p* < 0.05, *exposed vs. control.

We then compared the cytotoxicity of the three QDs, either pristine or aged, using two complementary assays, i.e., WST-1 assay, which probes cell metabolic activity, and LDH assay, which probes cell membrane integrity. Among the pristine QDs, only the grad QD showed significant cytotoxicity at the highest concentration, i.e., 100 nM, both in the WST-1 and in the LDH assays (Figures 3A,B). In contrast, aged QDs showed significant cytotoxicity at concentrations higher than 6.25 nM (thick) or 12.5 nM (grad and thin) (Figures 3C,D). At all concentrations, the aged thick QD showed similar or slightly greater cytotoxicity than the aged grad and aged thin QDs. We previously showed that InZnP QDs degraded when exposed to UV light, leading to the release of Zn(II) and In(III) ions. These ions could be responsible for the QDs' cytotoxicity. Since all three QDs contained the same amount of In but the thick QD contained more Zn than the grad and thin QDs, we hypothesized that Zn(II) ions released from QDs would explain the higher cytotoxicity of aged thick QD. To test this hypothesis, we evaluated the cytotoxicity of indium(III)-acetate and zinc(II)-acetate, also using WST-1 and LDH assays (Figures 3E,F). Only Zn(II)-acetate showed significant toxicity at the highest concentrations, i.e., up from 100 μM. This concentration is greater than the amount of Zn(II) contained in QDs [50 nM of thick QD contains 3.75 μM of In(III) and 15 μM of Zn(II)], which suggests that Zn(II) could play a role in the cytotoxicity of aged QDs, but that it is not the only determinant of their cytotoxicity.

### Oxidative Stress and Inflammation

**Figure 4:**
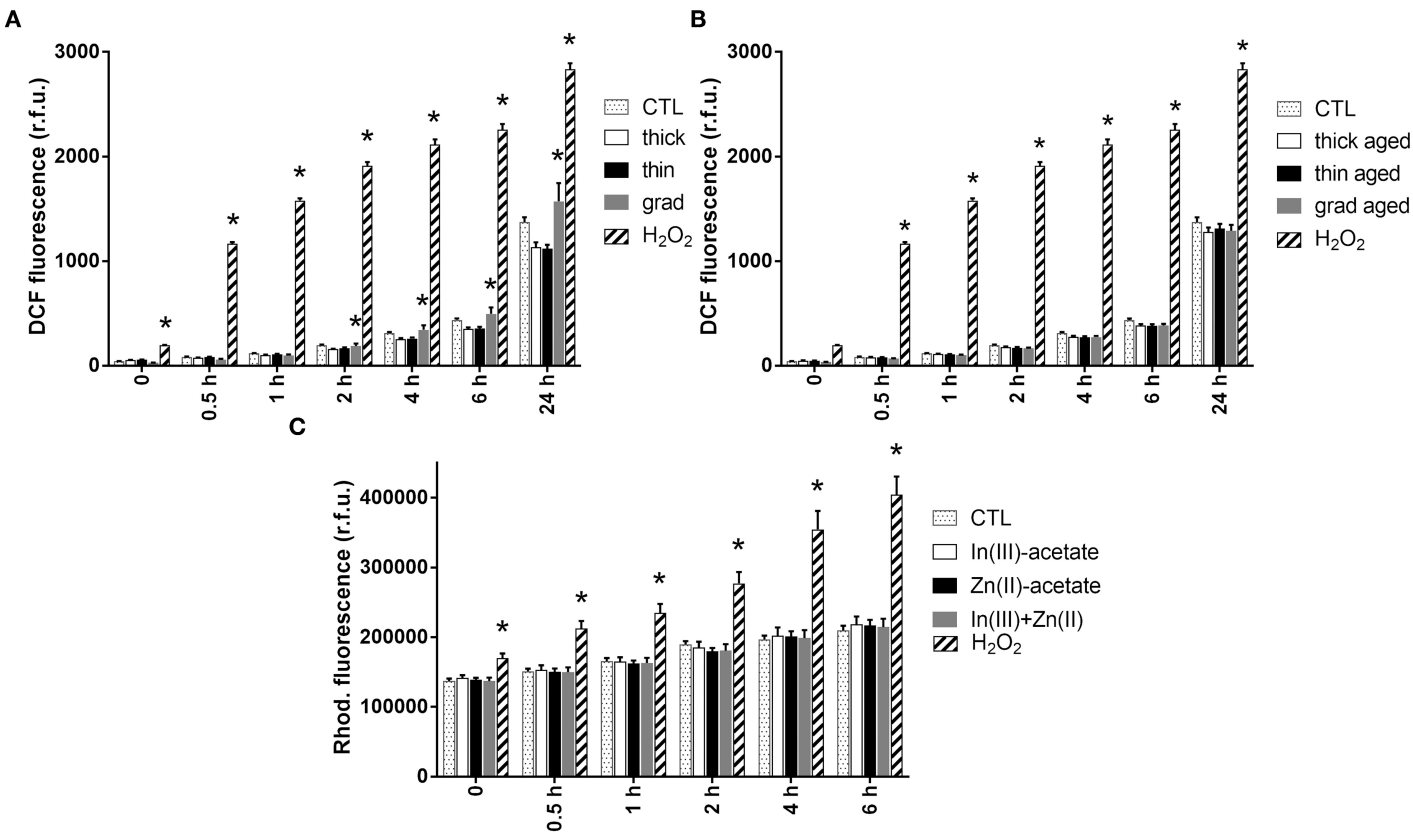
Reactive oxygen species (ROS) content in keratinocytes exposed to InZnP quantum dots (QDs), indium, and/or zinc acetate. ROS content was assessed via the 2′,7′-dichlorodihydrofluorescein diacetate (H2DCFDA) assay (QDs) and dihydrorhodamine 123 dye (DHR 123) assay [In(III)-acetate and Zn(II)-acetate]. Cells were exposed 24 h to 50 nM of pristine QDs **(A)**; 12 nM of aged QDs **(B)**; 3.75 μM of In(III)-acetate (In-ac), 15 μM of Zn(II)-acetate (Zn-ac), or a mixture of both 3.75 μM of In(III)-acetate and 15 μM of Zn(II)-acetate (In + Zn) **(C)**. Positive control: 500 μM of H_2_O_2_. Data are mean ± standard deviation of three independent experiments, performed on human primary keratinocytes from two different donors, with five replicates per experiment. Statistical significance: *p* < 0.05, *exposed vs. control.

Since the main mechanisms of InP/ZnS QD toxicity reported to date are via oxidative stress and inflammation (Chen et al., 2018, 2019), we measured the intracellular ROS content in cells exposed to the three QDs, using 2′,7′-dichlorodihydrofluorescein diacetate (H2DCFDA). Pristine grad QD caused a slight and significant elevation of the intracellular ROS content from 2 h of exposure, which persisted up to 24 h of exposure (Figure 4A and Supplementary Figure 3). Conversely, no ROS accumulation was observed in cells exposed to the other pristine QDs (Figure 4A), nor with aged QDs (Figure 4B). In addition, neither Zn(II)-acetate nor In(III)-acetate caused any accumulation of ROS in keratinocytes (Figure 4C and Supplementary Figure 4).

**Figure 5:**
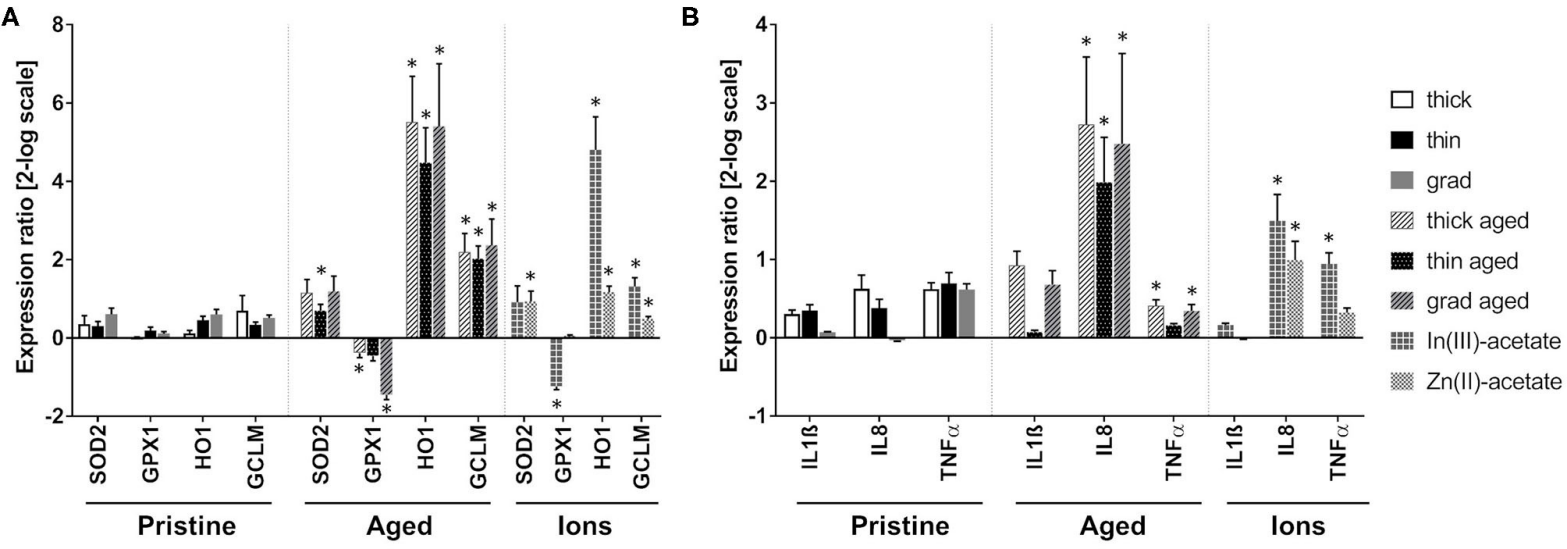
Antioxidant enzyme and inflammation marker mRNA expression modulation in keratinocytes exposed to pristine and aged InZnP quantum dots (QDs). Quantitative reverse transcription PCR (RT-qPCR) analysis of mRNA expression of antioxidant enzymes **(A)** and inflammation markers **(B)** in cells exposed for 24 h to 25 nM pristine QDs, 6.25 nM of aged QDs, 50 μM of In(III)-acetate, or 50 μM of Zn(II)-acetate. Data are mean ± standard deviation of three biological replicates (two technical replicates), on keratinocytes from a single human donor. Statistical significance: *p* ≤ 0.05, *exposed vs. control.

The mRNA expression of genes encoding antioxidant enzymes was quantified by RT-qPCR (Figure 5A). No significant modulation of the mRNA expression of superoxide dismutase 2 (SOD2), glutathione peroxidase (GPX1), heme oxygenase (HO-1), and glutamate–cysteine ligase (GCLM) was observed in cells exposed to pristine QDs. In cells exposed to all three aged QDs, mRNA expression of HO-1 and GCLM was highly increased. Moreover, the mRNA expression of SOD2 was increased in cells exposed to aged thin QD, and GPX1 mRNA expression was decreased in cells exposed to thick and grad QDs. Increased expression of HO-1 and GCLM was also observed in cells exposed to In(III)-acetate and Zn(II)-acetate, as well as decreased mRNA expression of GPX1 in cells exposed to In(III)-acetate and increased mRNA expression of SOD2 in cells exposed to Zn(II)-acetate. All these changes suggest that both aged QDs and In(III)-acetate and Zn(II)-acetate disturbed the oxidative balance in keratinocytes. SOD1 and catalase (CAT) mRNA expressions were also tested; they were not significantly modulated (Supplementary Table 3).

Finally, the mRNA expression of a set of markers of inflammation was also evaluated (Figure 5B). The mRNA expression of IL-1β, IL-8, and TNF-α did not significantly change in cells exposed to pristine QDs. Conversely, the mRNA expression of IL-8 was increased in cells exposed to the three aged QDs, while TNF-α was increased in cells exposed to thick and grad QDs. This suggests the onset of an inflammatory response. IL-8 and TNF-α mRNA expressions were also increased in cells exposed to In(III)-acetate and IL-8 mRNA expression in cells exposed to Zn(II)-acetate.

### Genotoxicity

**Figure 6:**
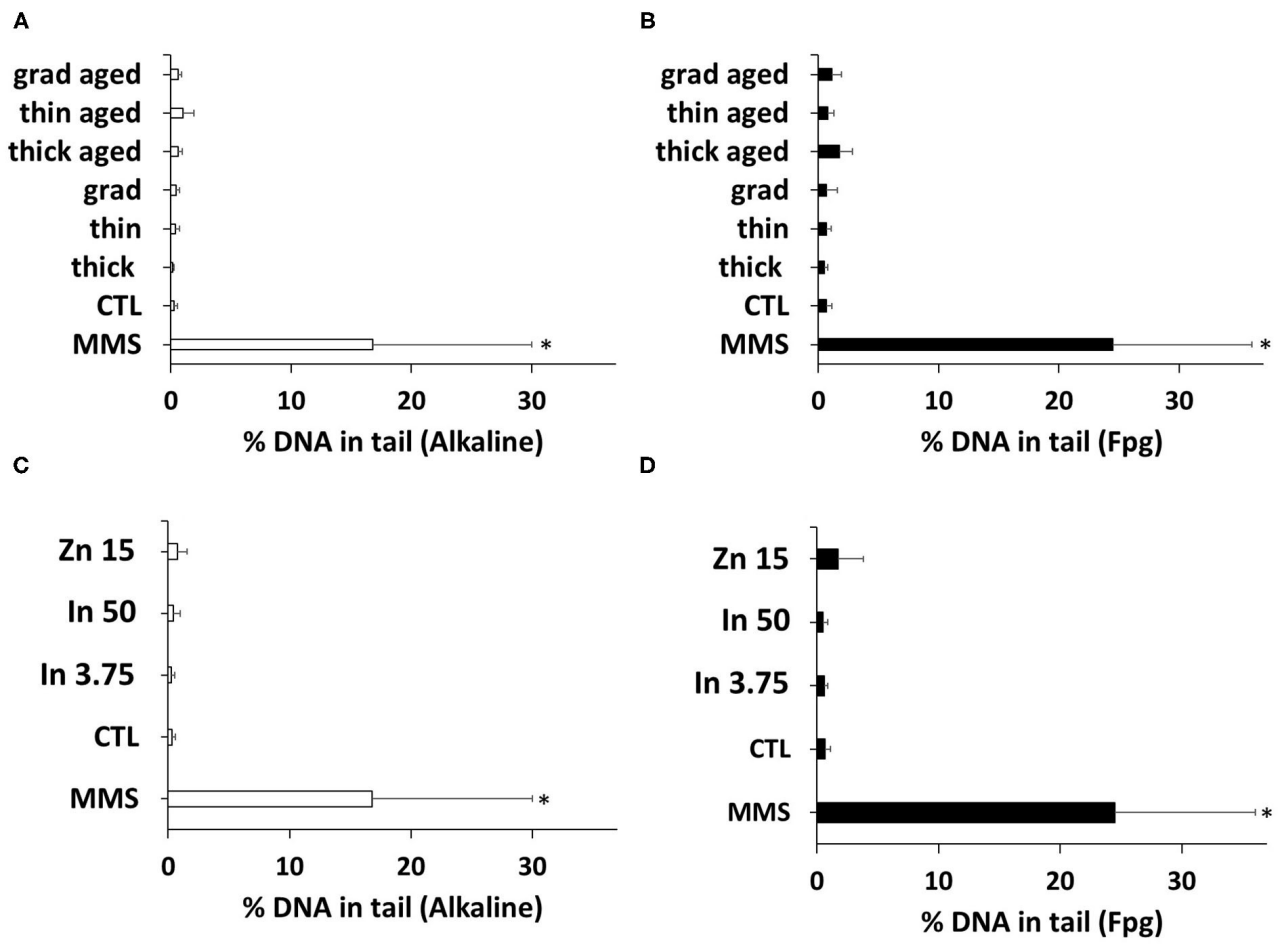
Quantum dot (QD) genotoxicity, assessed via the comet assay. DNA integrity was assessed via the alkaline **(A,C)** or the Fpg-modified comet assay **(B,D)**. Cells were exposed for 24 h to 25 nM of pristine QDs and 3 nM of aged QDs. Methyl methanesulfonate (MMS, 100 μM) was used as positive control. Data are mean ± standard deviation of six independent experiments, performed on human primary keratinocytes from three different donors, with two technical replicates per experiment. Statistical significance: *p* < 0.05, *exposed vs. control.

Since oxidative stress may trigger oxidative damage to DNA, we evaluated the impact of the three QDs on DNA integrity. Using the comet assay, both alkaline and Fpg-modified, no significant increase of DNA strand breaks, alkali-labile sites, and Fpg-sensitive sites was observed in keratinocytes exposed to either pristine or aged QDs or to In(III)-acetate or Zn(II)-acetate (Figure 6).

**Figure 7:**
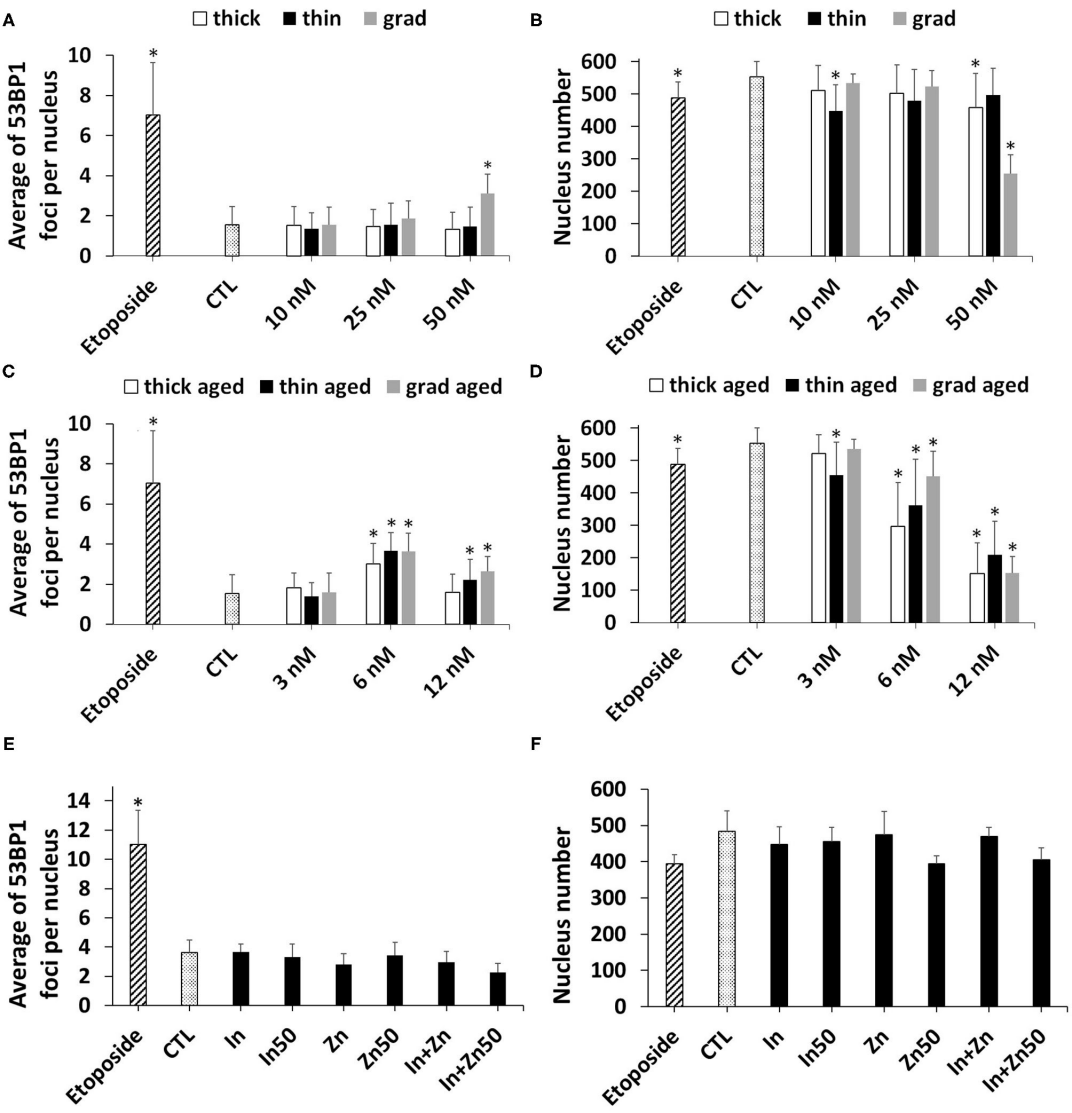
Quantum dot (QD) genotoxicity, assessed via 53BP1 immunostaining. Counting of 53BP1 foci **(A,C,E)** and of cell nuclei **(B,D,F)** via automated counting by HCA. Cells were exposed for 24 h to pristine QDs **(A,B)** or aged QDs **(C,D)**. Cells exposed to 3.75 nM (In) or 50 nM (In50) of In(III)-acetate, 15 nM (Zn) or 50 M (Zn50) of Zn(II)-acetate, a mixture of 3.75 μM of In(III)-acetate and 15 μM of Zn(II)-acetate (In + Zn), or a mixture of 50 μM of In(III)-acetate and 50 μM of Zn(II)-acetate (In + Zn50) **(E,F)**. Etoposide (100 μM) was used as a positive control. Data are mean ± standard deviation of three independent experiments, performed on human primary keratinocytes from two different donors, with five replicates per experiment. Statistical significance: *p* < 0.05, *exposed vs. control.

The presence of double-strand breaks was probed via 53BP1 immunostaining. Like γH2AX, it is an early biomarker of genotoxicity linked to DNA oxidations, which directly or indirectly leads to double-strand breaks (Panier and Boulton, 2014). Among pristine QDs, the grad QD induced a significant elevation of 53BP1 foci count, at the highest tested concentration, i.e., 50 nM (Figure 7A). This condition also significantly decreased the number of cells remaining in the wells after exposure (Figure 7B), i.e., some cells had been lost during the immunostaining procedure. Since this QD concentration was not cytotoxic as probed via the WST-1 and LDH assays (see *Cytotoxicity of Pristine and Aged QDs* section), we hypothesized that such condition would impair cell adherence to the plate. As a result, some of these loosely fixed cells were lost during the multiple washing steps of the immunostaining. As reported in Supplementary Figure 5, cells exposed to 50 nM of pristine grad QDs for 24 h adopted a rounded shape, confirming that their adherence to the plate was impaired. Exposure to 6 or 12 nM of the aged QDs, whatever their composition, also significantly increased the 53BP1 foci count as compared to control cells (Figure 7C). Again, the number of cells remaining in the wells was decreased, suggesting loss of cell adherence to the plate (Figure 7D). Finally, no significant increase of 53BP1 foci count was observed in cells exposed to In(III)-acetate or Zn(II)-acetate (Figure 7E). Such exposure did not induce any change in the number of cells remaining in the well (Figure 7F).

### Impact on Metal Homeostasis

**Figure 8:**
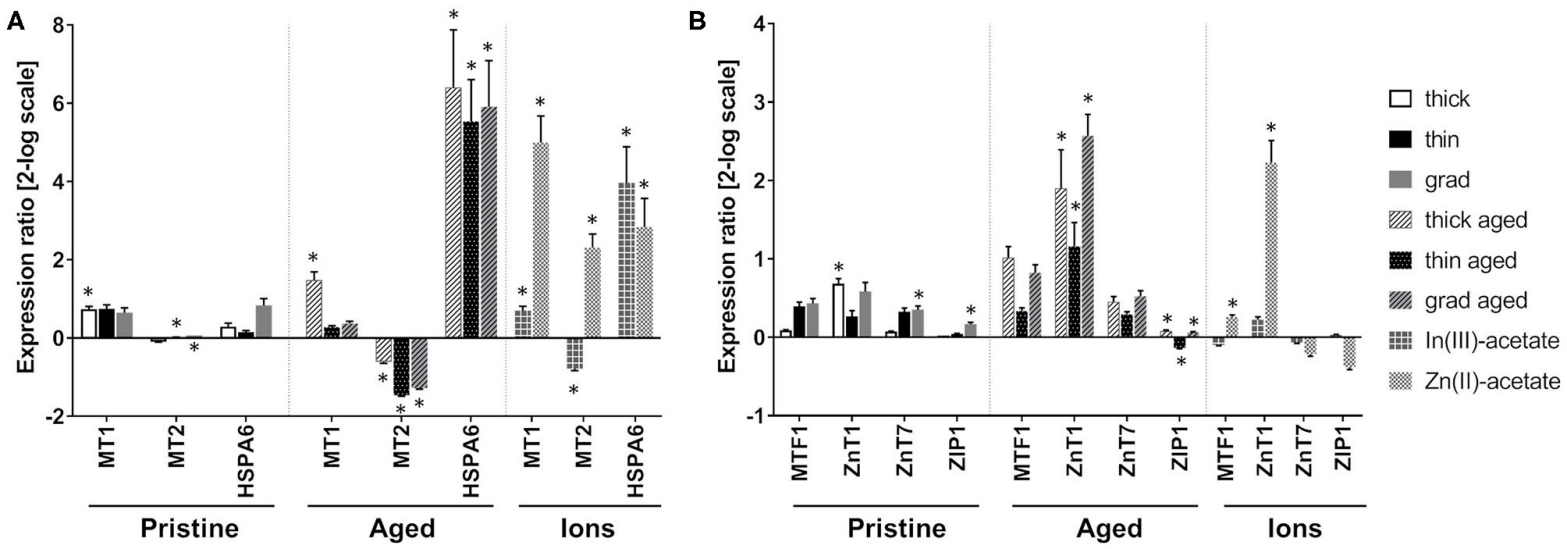
Impact of quantum dots (QDs) on metals homeostasis in keratinocyte. Quantitative reverse transcription PCR (RT-qPCR) assay analysis of the mRNA expression of genes encoding proteins involved in metal homeostasis **(A)** with a focus on Zn homeostasis **(B)**. Human primary keratinocytes were exposed for 24 h to 25 nM of pristine QDs, 6.25 nM of aged QDs, or 50 μM of In(III)-acetate or Zn(III)-acetate. Data are mean ± standard deviation of three independent experiment performed on human primary keratinocytes from the same donor, with two technical replicates. Statistical significance: *p* ≤ 0.05, *exposed vs. control.

These QDs contain In, Zn, and Se, all of which being metals or metalloids that can be potentially toxic to cells. To assess any cellular stress that could be due to metals, we analyzed the mRNA expression of metallothionein 1 and 2 (MT1 and MT2) (Figure 8A). These two proteins are involved in the overall regulation of metal homeostasis in cells, as well as in the regulation of cell oxidative status. Pristine thick QD increased the mRNA expression of MT1, while pristine thin and grad QDs mildly increased and decreased the mRNA expression of MT2, respectively. Stronger mRNA expression changes were observed in cells exposed to aged QDs. Marked increase of MT1 and decrease of MT2 mRNA expressions were observed in cells exposed to aged thick QD and to the three aged QDs, respectively (Figure 8A). This differs from the typical response to divalent metal ions, which classically leads to increased mRNA expression of both MT1 and MT2 (Emri et al., 2015). Still, MT1 and MT2 mRNA expression changes depend on the metal ion that triggers the metallic stress (Murata et al., 1999; Sims et al., 2012). For this reason, we hypothesized that the particular profile observed in cells exposed to aged QDs could result from a response to In(III) ions. To test this hypothesis, we measured the mRNA expression of MT1 and MT2 in cells exposed to In(III)-acetate and Zn(II)-acetate. Similar increased MT1 mRNA expression and decreased MT2 mRNA expression were observed in cells exposed to In(III)-acetate, while typical increase of MT1 and MT2 mRNA expressions was observed in cells exposed to Zn(II)-acetate (Figure 8A), which confirms our hypothesis. Finally, the mRNA expression of HSPA6, which is upregulated in response to a variety of cellular stresses, was strongly increased in cells exposed to the aged QDs, In(III)-acetate and Zn(II)-acetate (Figure 8A).

Furthermore, as shown in the *Cytotoxicity of Pristine and Aged QDs* section, the aged thick QDs were slightly more cytotoxic than aged thin and grad QDs. Since their composition differs only in their ZnS content, which is higher in thick QDs than in thin and grad QDs, we hypothesized that Zn(II) ions that are potentially released from QDs upon aging (Tarantini et al., 2019) could play a role in their cytotoxicity. To test this hypothesis, the mRNA expression of proteins involved in intracellular zinc homeostasis regulation was assessed by RT-qPCR. We evaluated the expression of ZnT1 and ZnT7, which are responsible for the export of Zn(II) out of the cell and its storage in the Golgi apparatus, respectively, and of ZIP1, which imports Zn(II) inside the cell. The mRNA expression of MTF1, which is a Zn-dependent transcriptional regulator involved in cellular response to heavy metals, particularly in the regulation of MT1 and MT2 expression was also evaluated. Pristine QDs only induced mild modulation of the mRNA expression of some of these proteins, i.e., ZnT1 in cells exposed to the thick QD as well as ZnT7 and ZIP1 in cells exposed to the grad QD (Figure 8B). Conversely, all three aged QDs induced much stronger modulation of the mRNA expression of these proteins, with a strong increase of ZnT1 mRNA expression, as well as a mild but significant ZIP1 mRNA expression change, which was increased in cells exposed to thick and grad QDs and decreased in cells exposed to the thin QD (Figure 8B). Increased mRNA expression of MTF1 and ZnT1 was also observed in cells exposed to Zn(II)-acetate (Figure 8B).

## Discussion

In this study, we show that three InZnP core/shell QDs, developed to be safer-by-design, induce a toxic response in human primary keratinocytes that depends on the composition of the QD. It also depends on their physicochemical state, i.e., pristine vs. aged in environmentally relevant weathering conditions. In brief, the two pristine double-shell QDs do not cause any adverse outcome in keratinocytes, while the pristine gradient shell QD causes cytotoxicity at the highest tested concentration and increases the intracellular ROS content and the number of 53BP1 foci, reflecting oxidative damage to DNA. In contrast, all three aged QDs show more intense toxicity, with significant cytotoxicity, genotoxicity, perturbation of cell redox metabolism, and inflammatory response. Both pristine and aged QDs significantly dysregulate metal homeostasis and particularly Zn homeostasis, with aged QDs dysregulating it more intensely than pristine QDs.

When considering the cytotoxicity of these nanocrystals, measurement of keratinocyte metabolic activity with the WST-1 assay and of membrane damage with the LDH leakage assay leads to the same result, i.e., significant and higher toxicity of aged QDs as compared to pristine QDs. In our earlier study using InZnP/Zn(Se,S) QDs having different composition, we correlated such a behavior to the release of toxic elements from the core of the QDs due to the photodegradation of the InZnP and InZnSP structure (Tarantini et al., 2019). Moreover, the oxidation of InP/ZnS QDs has been shown to be accelerated by lysosomal condition and to lead to their dissolution and to the release of indium and zinc ions (Brunetti et al., 2013). This would explain why among the three pristine QDs only the QD with a single gradient shell shows significant toxicity, because it is the least protected from lysosomal conditions when accumulated in cells. This shows that our safer-by-design strategy, consisting in coating core–shell InP QDs with a second shell of ZnS, was efficient and effectively reduced their toxicity. These findings are also in line with the results reported by Chibli et al. (2011), demonstrating reduced cytotoxicity of double-shell InP/ZnS QDs, as compared to single-shell InP/ZnS QDs. We observe loss of cell viability after exposure for 24 h to 100 nM of InZnP/Zn(Se,S) grad QD. For comparison, Soenen et al. (2014) reported the onset of cell mortality in cells exposed for 24 h to concentrations of InP/ZnS QDs higher than 80 nM. Chen et al. (2018) did not observe any loss of cell viability upon exposure to up to 160 μg/ml InP/ZnS QDs for 24 h, and Chibli et al. (2011) observed cell mortality when exposed to 100–500 nM InP/ZnS QDs, depending on the tested cell line. In the two latter studies, the use of a different metrics and different exposure conditions makes it difficult to compare the results to the one reported here. Overall, the cytotoxicity observed here with InZnP/Zn(Se,S) and InZnP/Zn(Se,S)/ZnS QDs is comparable to that observed for InP/ZnS QDs. Still, the presence of Zn in the core of these QDs enhances their fluorescence, which in a safer-by-design perspective confers an advantage to the present formulations compared to InP/ZnS QDs. In addition to cytotoxicity evaluation via the WST-1 and LDH assay, in the course of the 53BP1 assay, we observed that exposure to InZnP QDs led to cell detachment from the plates. Indeed, while the number of cell nuclei before and after the 53BP1 staining was constant in control conditions, it was reduced drastically in the wells where the cells had been exposed to QDs. Moreover, optical microscopy observation showed that cells adopted a round shape after exposure to QDs. A hypothesis to explain this cell detachment is that exposure to QDs decreases the cell adhesion property, either via altering surface proteins that are responsible for cell adhesion to the plastic dish, such as cell adhesion molecules (CAMs), or via disturbing the cell cytoskeleton. This is another characteristic of InP QD toxicity that would need to be explored.

Importantly, our results show that capping the InP-based QDs with an additional ZnS shell on top of the gradient Zn(Se,S) shell is not sufficient to completely preclude QDs' degradation in harsh environmental conditions (the aging conditions used here simulate exposure to full-spectrum sunlight on a sunny day at noon). It results in QDs that totally degrade and release potentially toxic In(III), Zn(II), and selenium ions or their oxidation products. Importantly, this second shell made of ZnS increases the QD Zn content, and our results suggest that Zn plays a major role in the toxicity of these QDs. Moreover, the cytotoxicity of the aged thick QD, which contains the highest level of Zn, is slightly higher than that of grad and thin shell QDs. Due to its pleiotropic function in cells (Maret, 2019), the intracellular Zn content is finely tuned with concentrations not exceeding a few picomolars (Simons, 1991). Exposure of cells to Zn-containing nanoparticles, such as the QDs tested in the present study, results in the accumulation of Zn in cells by endocytosis (Oh and Park, 2014), i.e., following a much less-controlled entry route than the physiological accumulation of Zn via the ZIP1 transporter. Consequently, cells experience Zn overload, which is detrimental to their proper function (Maret, 2019). The cellular response to exposure to aged QDs, characterized by perturbation of the oxidative balance and Zn homeostasis, particularly with the activation of the MTF1 transcription factor that further induces mRNA expression of metallothioneins and ZnT transporters, is a typical response to a Zn overload (Emri et al., 2015). Noteworthy, the variation of MT1 and MT2 mRNA expression observed in cells exposed to QDs differs from the typical response to Zn exposure, which is characterized by upregulation of both MT1 and MT2 (Emri et al., 2015). The regulation of MT expression is acknowledged to depend upon the ions to which cells have been exposed (Murata et al., 1999; Sims et al., 2012). Here, we observe upregulation of MT1 and downregulation of MT2, as also observed in cells exposed to In(III)-acetate. The cellular response to indium ions has been documented poorly so far; this transcriptional profile of MT1 and MT2 could be a typical feature of the cell response to this heavy metal. Therefore, we can infer that our strategy of coating InPZn QDs with a double shell of zinc chalcogenides is a promising safer-by-design strategy, because it reduces the cytotoxicity of pristine QDs. However, it does not deliver safe-by-design QDs, as aged QDs are still much more toxic than pristine QDs and particularly aged thick QDs.

In addition to these toxicity outcomes, aged InZnP QDs and Zn^2+^ and In^3+^ ions significantly modulate the mRNA expression of antioxidant enzymes, although no significant modulation of intracellular ROS content is observed. This indicates a perturbation of the oxidative balance within the cells, to which the cell responds efficiently. Such alteration of the cell redox status after exposure to InP QDs has been reported by others in lung cells, neural progenitor cells, vascular endothelial cells, and in immune cells (Soenen et al., 2014; Chen et al., 2018, 2019; Ayupova et al., 2019). Chen et al. (2019) link this stress with the onset of inflammatory conditions, which is also suggested in the present study by the dysregulation of mRNA expression of IL-8 and TNF-α in cells exposed to aged InZnP QDs as well as In(III) and Zn(II) ions. Importantly, while oxidative stress is often linked to oxidative damage to DNA, only one article focuses on InP/ZnS QD genotoxicity and reports no significant outcome in the γ-H2AX assay in cells exposed to these QDs (Soenen et al., 2014). We also previously assessed oxidative stress and genotoxicity caused by a variety of InZnP QD formulations and observed no elevation of intracellular ROS content and no genotoxicity in the comet assay (Tarantini et al., 2019), which is in line with the findings of the present study. However, in the present study, InZnP QDs show significant genotoxicity via inducing some 53BP1 foci. The comet assay in its alkaline version makes possible the detection of DNA single-strand and double strand breaks and alkali-labile sites, such as abasic sites. It is less specific than the 53BP1 assay, which mainly probes the presence of DNA double-strand breaks. Higher specificity can explain the higher sensitivity of the 53BP1 assay to double-strand breaks, as compared to the comet assay. Indeed, in the comet assay, a few double-strand breaks cannot be discriminated from the background level of DNA lesions that continuously occur in the cells and are repaired. This explains why we observe significant genotoxicity via the 53BP1 assay but not via the comet assay. This underlines the need to combine several complementary tests in order to conclude definitely on a genotoxic effect of NPs. Genotoxicity of indium, in distinct speciation as compared to its speciation in present study, has been reported in the literature. Not only indium oxide and indium tin oxide particles and nanoparticles but also indium chloride have been reported to be genotoxic via the comet assay, micronucleus assay, and measurement of 8-nitroguanine DNA lesion (Tabei et al., 2018; Ahmed et al., 2020; Tsai et al., 2020). While Tsai et al. (2020) attribute indium-related genotoxicity to oxidative stress and mitochondrial dysfunction, Ahmed et al. (2020) rather attribute it to inflammation that triggers 8-nitroguanine formation in the DNA of exposed cells. Tabei et al. (2018) demonstrate that indium genotoxicity is not linked with oxidative stress but that it can be related to intracellular degradation of the indium-containing particles, which leads to accumulation of In(III) ions in the nuclei of exposed cells and direct interaction of indium with the DNA. Our experiments show no evidence of In(III) ion accumulation inside the nuclei of exposed cells, but EDX may not be sensitive enough to detect the presence of traces of In in 100-nm-thick sections of cell nuclei. Moreover, In(III) ions could have been washed out of cell nuclei during the sample preparation for STEM-EDX analysis. We rather observed QDs accumulated in the cell cytoplasm, very close to nuclei, which sometimes showed deformation due to the presence of these voluminous agglomerates. Such deformation has already been observed in cells exposed to TiO_2_ NPs and has been suggested as a possible mechanism for their genotoxicity (Di Virgilio et al., 2010; Magdolenova et al., 2014). Overall, multiple indirect mechanisms can explain NP genotoxicity. Indirect genotoxicity can result from interaction of NPs with proteins involved in DNA replication, transcription, or repair; with cell cycle checkpoints; with the mitotic spindle; bu also from production of ROS or Fenton-like reactions at the surface of the NP (Magdolenova et al., 2014). The mechanisms of InZnP QD genotoxicity would need to be clarified via additional experiments.

Such localization of QD as agglomerates at the vicinity of cell nuclei has been observed already in earlier reports (Chibli et al., 2011; Brunetti et al., 2013). It suggests that QDs accumulated inside cells via endocytosis, as reported earlier (Soenen et al., 2014), endocytosis being the main route of entrance of NPs inside cells (Francia et al., 2020). Noteworthy, the surface of these QDs has not been engineered to facilitate their cellular internalization by a specific entry route. These QDs are coated with penicillamine, on which a protein corona must have formed due to interaction with serum in the exposure medium. Given the size of the QD agglomerates observed inside the cells by TEM, we can speculate that they are internalized in cells via macropinocytosis (Francia et al., 2020). We did not observe any QDs inside the endoplasmic reticulum, Golgi, or any other organelle. We also did not observe any membrane surrounding QD agglomerates, suggesting either that the membrane was very tightly bound to the surface of the QD agglomerate or that the QDs had escaped the endosomes. So far, endosomal escape has been the matter of intense research in nanomedicine and it is still considered as very inefficient (Martens et al., 2014). Therefore, it is improbable that the InZnP QDs could escape endosomes. We rather speculate that the intracellular agglomerates of QDs are surrounded by a membrane, which is not visible in our TEM images because of a too low contrast or because it very closely packs the NP agglomerates.

## Conclusion

In this study, we evaluated the toxicity of InP QDs developed to be safer by design, both in their pristine state and after environmentally relevant degradation, i.e., after accelerated weathering in a climatic chamber. We show that pristine QDs are mildly toxic and that their toxicity is reduced when the design of their shell is improved by increasing its thickness, hence its resistance to UV light. Conversely, aged QDs are more toxic, causing loss of cell viability, damage to DNA, deregulation of the oxidative balance in exposed cells, as well as inflammation. More importantly, we show that cell exposure to aged QDs modulates the expression of a series of markers of intracellular zinc homeostasis, suggesting that Zn plays a major role in their toxicity. Moreover, the change in expression of metallothioneins follows a typical pattern of exposure to In(III) ions, suggesting that In also plays a role in the toxicity of these QDs. Therefore, while our safer-by-design strategy is promising, further improvements are needed, for instance, by designing more robust shells to counteract the effects of aging.

## Data Availability Statement

The raw data supporting the conclusions of this article will be made available by the authors, without undue reservation.

## Ethics Statement

Studies involving animal subjects: No animal studies are presented in this manuscript. Studies involving human subjects: No human studies are presented in this manuscript. Inclusion of identifiable human data: No potentially identifiable human images or data is presented in this study.

## Author Contributions

FD performed QD aging experiments and all cell biology experiments. KW synthesized the QDs. CM and BG prepared and imaged the cells by electron microscopy. P-HJ performed STEM and EDX analyses. PR and MC conceived and supervised the whole study. FD and MC wrote the manuscript. All the authors edited the manuscript and approved its submission.

## Conflict of Interest

The authors declare that the research was conducted in the absence of any commercial or financial relationships that could be construed as a potential conflict of interest.
