# Supplementary material for: Evaluation of the Dermal Toxicity of InZnP Quantum Dots Before and After Accelerated Weathering: Toward a Safer-By-Design Strategy
Source: Front Toxicol. 2021 Mar 22;3:636976. doi: 10.3389/ftox.2021.636976 (PMC8915823; doi:10.3389/ftox.2021.636976)
Supplement: Supplementary file 1 [file Table_1.DOCX]

**Supplementary material for:**

Mechanisms of toxicity of safer-by-design InZnP QDs, both pristine and after ageing, using an environmentally-relevant end-of-life scenario

By: Fanny Dussert, Karl David Wegner, Christine Moriscot, Benoit Gallet, Pierre Henry Jouneau, Peter Reiss, Marie Carrière

**Table S1. Photophysical and structural properties of QDs**

|  | **Gradient shell** | **Thin shell** | **Thick shell** |
| --- | --- | --- | --- |
| 1^st^ exciton peak (nm) | 518 | 518 | 518 |
| PL maxima | 578 | 568 | 564 |
| FWHM | 66.2 | 61.3 | 60.0 |
| In (%at.) | 1 | 1 | 1 |
| P (%at.) | 1.1 | 0.9 | 1 |
| Zn (%at) | 1.9 | 5.1 | 8.6 |
| Se (%at) | 0.5 | 1 | 1.2 |
| S (%at) | 2 | 5 | 9.1 |
| Core diameter | 2.67±0.32 nm | 2.67±0.32 nm | 2.67±0.32 nm |
| Core-shell diameter | 3.6±1.0 nm | 4.1±0.8 nm | 6.2±1.0 nm |
| Nr. of shell layers | 2 monolayers | 3 monolayers | 4-5 monolayers |
| Z-average, growth medium (PdI) | 565 nm (0.645) | 151 nm (0.346) | 467 nm (0.820) |
| Zeta potential in PBS | -21 mV | -22 mV | -21 mV |
| Zeta potential in growth medium | -7 mV | -8 mv | -8 mV |

*^a^* Photophysical data and EDX data are have been published in a previous article ([Wegner et al., 2019](#_ENREF_1)). Core and core-shell diameters were measured from cryo-TEM images, acquired on a FEI Polara microscope operating at 300 kV and recorded on a Gatan K2 camera. Number of shell layers (Nr. shell layers) was deduced from EDX data. DLS and zeta potential measurements were recorded on a zetasizer Nano Series (Malvern).

**
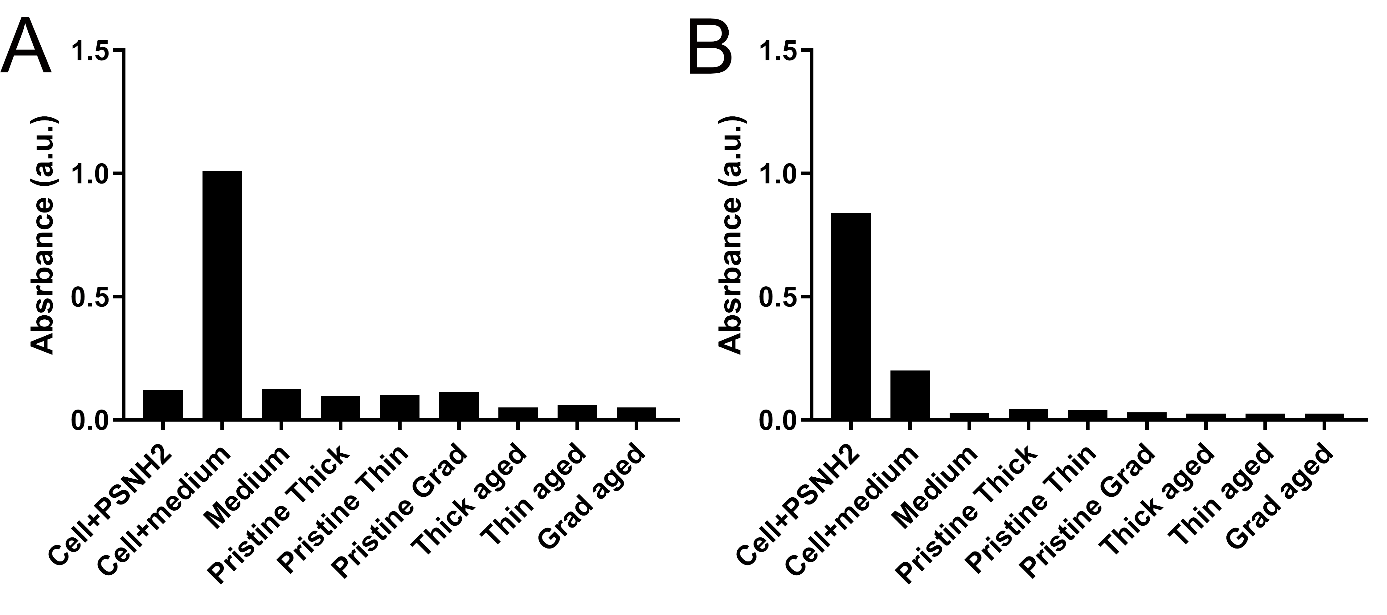
**

**Fig. S1.** Interference of QDs with cytotoxicity assays. Interference of QDs with the chemical reaction of WST1 (A) and LDH (B) assays was assessed. (A) shows the absorbance measured after incubating for 90 min with WST-1 cells exposed for 24 h to 100 µg/mL PS-NH_2_ (Cell+PSNH2) and control cells exposed for 24 h to cell culture medium (Cell+medium). Absorbance was also measured after incubating WST-1 for 90 min with cell culture medium (medium) or 100 nM of QDs (pristine Thick, Thin and Grad, and aged Thick, Thin and Grad) without any cells. (B) represents the absorbance after the LDH reaction of exposure medium of cells exposed for 24 h to 100 µg/mL PS-NH_2_ (Cell+PSNH2) and control cells exposed for 24 h to cell culture medium (Cell+medium). It also shows absorbance of QDs when incubated for 30 min at room temperature with LDH reagent.

**Table S2.** Primers sequences used in RT-qPCR experiments.

|  | **Forward** | **Reverse** |
| --- | --- | --- |
| CycloA | 5’-TTC-ATC-TGC-ACT-GCC-AAG-AC-3’ | 5’-TCG-AGT-TGT-CCA-CAG-TCA-G-3’ |
| S18 | 5’-AAC-GTC-TGC-CCT-ATC-AAC-TTT-3’ | 5’-TGG-ATG-TGG-TAG-CCG-TTT-TCT-3’ |
| GADPH | 5’-GAG-TCA-ACG-GAT-TTG-GTC-GT-3’ | 5’-TTG-ATT-TTG-GAG-GGA-TCT-CG-3’ |
| SOD1 | 5’-AGG-GCA-TCA-TCA-ATT-TCG-AG-3’ | 5’-ACA-TTG-CCC-AAG-TCT-CCA-AC-3’ |
| SOD2 | 5’-TCC-ACT-GCA-AGG-AAC-AAC-AG-3’ | 5’-TCT-TGC-TGG-GAT-CAT-TAG-GG-3’ |
| GPX1 | 5’-CCA-GTC-GGT-GTA-TGC-CTT-CT-3’ | 5’-CTC-TTC-GTT-CTT-GGC-GTT-CT-3’ |
| CAT | 5’-AGC-TTA-GCG-TTC-ATC-CGT-GT-3’ | 5’-TCC-AAT-CAT-CCG-TCA-AAA-CA-3’ |
| HO-1 | 5’-TTC-TCC-GAT-GGG-TCC-TTA-CAC-T-3’ | 5’-GGC-ATA-AAG-CCC-TAC-AGC-AAC-T-3’ |
| MT1 | 5’-GCT-TCT-CCT-TGC-CTC-GAA-3’ | 5’-TGA-CGT-CCC-TTT-GCA-GAT-3’ |
| MT2 | 5’-TGC-ATT-TGC-ACT-CTT-TGC-AT-3’ | 5’-TCT-TCA-GCT-CGC-CAT-GGA-T-3’ |
| ZnT1 | 5’-CGC-AGC-CAT-GGG-GTG-TTG-GG-3’ | 5’-CAT-CGC-CAG-CGA-CGA-GGT-CA-3’ |
| ZnT2 | 5’-AAC-TTT-GGC-TGG-CAG-AGA-GC-3’ | 5’-TGG-TGA-AGG-GTC-AAC-CCC-AT-3’ |
| ZnT7 | 5’-GTG-CCT-GAA-CCT-CTC-TTT-CG-3’ | 5’-CGG-AAA-TCA-GCC-TAA-GCA-G-3’ |
| ZIP1 | 5’-GCC-AGG-AGC-TAA-CCA-TGA-AG-3’ | 5’-ATG-GCC-AGG-ATG-AAC-TCT-TG-3’ |
| MTF1 | 5’-CGA-AGG-AGA-AGC-CAT-TTG-AG-3’ | 5’-ATT-TGC-TGC-AGC-CTT-CAG-AT-3’ |
| HSPA6 | 5’-AGG-AGG-TGG-AGA-GGA-TGG-TT-3’ | 5’-TGT-CCT-CTT-CGG-GAA-TCT-TG-3’ |
| GCLM | 5’-AGT-CCT-TGG-AGT-TGC-ACA-GC-3’ | 5’-ACA-CAG-CAG-GAC-GCA-AGA-TT-3’ |
| IL1β | 5’-ACA-GAT-GAA-GTG-CTC-CTT-CCA-3’ | 5’-GTC-GGA-GAT-TCG-TAG-CTG-GAT-3’ |
| TNFα | 5’-GAG-CAG-TGA-AAG-CAT-GAT-CC-3’ | 5’-CGA-GAA-GAT-GAT-CTG-ACT-GCC-3’ |
| IL8 | 5’-GAA-TGG-GTT-TGC-TAG-AAT-GTG-ATA-3’ | 5’-CAG-ACT-AGG-GTT-GCC-AGA-TTT-AAC-3’ |
|  |  |  |

**Fig. S2.** TEM image of keratinocytes exposed to aged thick QD. Cells were exposed for 24 h to 3 nM of aged thick QD, then fixed with paraformaldehyde and glutaraldehyde, post-fixed with osmium tetroxide, cut and observed via transmission electron microscopy without any counterstaining. Electron-dense precipitates were observed in the cell cytoplasm (see insert for a more focused image).

**Fig. S3.** Accumulation of reactive oxygen species into keratinocytes after exposure to the InZnP QDs. ROS measurement was assessed via H_2_DCFDA assay for QDs exposure and cells were exposed 24 h to 25 or 10 nM of pristine (A-B) and 6 or 3 nM of aged QDs (C-D). H_2_O_2_ (500 µM) or KBrO_3_ (1 mM) were used as positive controls. Graphs represent mean +/- standard deviation of two independent experiments, performed on human primary keratinocytes from different donors, with 5 replicates per experiment. Statistical significance: p < 0.05, *: exposed vs. control.

**Fig. S4.** Accumulation of reactive oxygen species into keratinocytes after exposure to In-III)-acetate and/or Zn(II)-acetate. ROS accumulation was assessed via the DHR 123 assay on cells were exposed for 24 h to 50 µM of In(III)-acetate, 50 µM of Zn(II)-acetate or a mixture of both. H_2_O_2_ (500 µM) was used as a positive control. Graphs represent mean +/- standard deviation of two independent experiments, performed on human primary keratinocytes from different donors, with 5 replicates per experiment. Statistical significance: p < 0.05, *: exposed vs. control.

**Table S3.** Summary of mRNA expressions in keratinocytes exposed to pristine or aged QDs*^a^*

|  | Thick | | Thin | | Grad | | Aged Thick | | Aged Thin | | Aged Grad | | In(III)-acetate | | Zn(II)-acetate | |
| --- | --- | --- | --- | --- | --- | --- | --- | --- | --- | --- | --- | --- | --- | --- | --- | --- |
|  | mean | sd | mean | sd | mean | sd | mean | sd | mean | sd | mean | sd | mean | sd | mean | sd |
| SOD1 | 0.298 | 0.177 | 0.045 | 0.011 | 0.265 | 0.044 | 0.449 | 0.100 | 0.350 | 0.061 | -0.432 | 0.180 | 0.490 | 0.065 | -0.029 | 0.003 |
| SOD2 | 0.353 | 0.218 | 0.309 | 0.111 | 0.606 | 0.154 | 1.158 | 0.335 | 0.700* | 0.156 | 1.193 | 0.388 | 0.917 | 0.414 | 0.936* | 0.261 |
| CAT | 0.527 | 0.302 | 0.324 | 0.106 | 0.546 | 0.172 | 0.873 | 0.272 | 0.179 | 0.055 | 1.066 | 0.393 | 0.602 | 0.130 | 0.151 | 0.021 |
| GPX1 | 0.015 | 0.011 | 0.194 | 0.082 | 0.113 | 0.050 | -0.383* | 0.115 | -0.454 | 0.128 | -1.450* | 0.120 | -1.235* | 0.084 | 0.057 | 0.021 |
| HO1 | 0.119 | 0.078 | 0.457 | 0.103 | 0.601 | 0.129 | 5.518* | 1.162 | 4.478* | 0.893 | 5.408* | 1.596 | 4.807* | 0.839 | 1.176* | 0.149 |
| GCLM | 0.697 | 0.389 | 0.335 | 0.075 | 0.514 | 0.073 | 2.195* | 0.474 | 2.028* | 0.323 | 2.374* | 0.662 | 1.318* | 0.222 | 0.490* | 0.059 |
| IL1ß | 0.069 | 0.011 | 0.346 | 0.076 | 0.305 | 0.048 | 0.927 | 0.179 | 0.075 | 0.021 | 0.682 | 0.177 | 0.163 | 0.024 | -0.017 | 0.002 |
| IL8 | -0.036 | 0.009 | 0.378 | 0.114 | 0.626 | 0.175 | 2.729* | 0.857 | 1.989* | 0.570 | 2.478* | 1.154 | 1.495* | 0.337 | 0.994* | 0.237 |
| TNFα | 0.618 | 0.071 | 0.695 | 0.139 | 0.621 | 0.082 | 0.410* | 0.076 | 0.160 | 0.022 | 0.346* | 0.078 | 0.945* | 0.139 | 0.322 | 0.056 |
| MTF1 | 0.090 | 0.007 | 0.394 | 0.055 | 0.433 | 0.062 | 1.016 | 0.141 | 0.339 | 0.038 | 0.826 | 0.099 | -0.097 | 0.011 | 0.259* | 0.027 |
| ZnT1 | 0.686* | 0.062 | 0.266 | 0.075 | 0.585 | 0.113 | 1.902* | 0.489 | 1.161* | 0.302 | 2.570* | 0.274 | 0.227 | 0.034 | 2.228* | 0.281 |
| ZnT7 | 0.075 | 0.004 | 0.326 | 0.045 | 0.352* | 0.047 | 0.453 | 0.067 | 0.295 | 0.032 | 0.525 | 0.070 | -0.067 | 0.009 | -0.215 | 0.026 |
| ZIP1 | -0.004 | 0.000 | 0.042 | 0.007 | 0.169* | 0.021 | 0.080* | 0.013 | -0.137* | 0.009 | 0.063* | 0.009 | 0.032 | 0.004 | -0.381 | 0.030 |
| MT1 | 0.738* | 0.071 | 0.742 | 0.106 | 0.648 | 0.122 | 1.484 | 0.205 | 0.281 | 0.033 | 0.374* | 0.058 | 0.704* | 0.107 | 4.998* | 0.676 |
| MT2 | -0.100 | 0.006 | 0.020* | 0.003 | -0.014* | 0.002 | -0.607* | 0.036 | -1.473* | 0.016 | -1.282* | 0.026 | -0.787* | 0.047 | 2.312* | 0.344 |
| HSPA6 | 0.296 | 0.086 | 0.151 | 0.038 | 0.836 | 0.173 | 6.406* | 1.468 | 5.537* | 1.069 | 5.912* | 1.179 | 3.967* | 0.924 | 2.838* | 0.728 |

*^a^* mRNA expression was quantified using RT-qPCR. Statistical significance: *p<0.05, exposed vs. control (unexposed cells), on n=3 biological replicates, and 2 technical replicates per biological replicate .

**Figure S5. Optical microscopy images of cells exposed to pristine grad QDs.** Control cells (A) and cells exposed to 50 nM of pristine grad QDs for 24 h. Magnification: x20.

**References:**

Wegner, K.D., Dussert, F., Truffier-Boutry, D., Benayad, A., Beal, D., Mattera, L., Ling, W.L., Carrière, M., and Reiss, P. (2019). Influence of the Core/Shell Structure of Indium Phosphide Based Quantum Dots on Their Photostability and Cytotoxicity. *Front Chem* 7**,** 466.
